# Dr. Ricord on Syphilis

**Published:** 1873-01

**Authors:** 


*Dr. Ricord on Syphilis.* (Meeting of the British Medical Association at Birmingham, August, 1872.)

Dr. Ricord, after acknowledging the reception which had been accorded him, said he had not prepared an address, as he had not come with the intention of speaking; but Mr. Acton had caught him and obliged him to speak, which was a trick. (Laughter.)

He had come to listen and to learn, but not to teach. However, he must say something, though there was no necessity for him to say much, as Mr. Acton had so nearly stated his views and his mode of treatment that there was very little for him to add. There was one great question in regard to syphilis, and it was this: could it be cured radically? In former times all venereal affections, no matter what, were considered as belonging to syphilis, and certainly there was then an immense number of radical cures by mercury or any other means. In this way swellings of the glands, soft chancres, even warts, and other things not belonging to syphilis, were easily enough cured, radically cured; and there were no after-consequences, no secondary symptoms. This explanation would account for the immensely large number of cases of (reputed) syphilis which used to be radically cured. But, since syphilis had been correctly diagnosed, the inquiry to which he had devoted a large part of his life was to see what belonged to syphilis, and what resembled it without belonging to it. There had been great differences in the results of treatment—so much so that a doubt, as Mr. Acton had said, had arisen whether real syphilis could be cured. That doubt as to the curability of syphilis was not recent; it was a doubt which old authors had expressed; and one particularly, with a curious name, which they would probably remember—"Mercurialis"—thought that now and then an armistice might probably be made with syphilis, but that there was no real cure. In fact, they frequently saw that a long time—months, years—after the symptoms had been treated, new symptoms appeared. And so the doubt whether syphilis could be radically cured, or whether the cure was only temporary, with a prospect of the symptoms returning, might still remain; he (Ricord), however, had established the law of the unicity of the diathesis of syphilis. The law of syphilis was the same as the law of small-pox, cow-pox, or measles. A man could have but one attack so long as the disease remained in the constitution—that was to say, according to his opinion a new attack could not take place while the system was still under the influence of the old diathesis. Well, it was exactly so with syphilis; as long as a patient was laboring under the diathesis of syphilis, another *infection* of syphilis could not occur—it was impossible. For instance, after indurated chancre, and the appearance of secondary symptoms, it was not possible for the patient to contract a new indurated chancre, with swelling of the glands, manifestation of skin disease, and so on. After one attack the patient could not have another infection as long as the influence of the first remained in his body; a second contagion could not take possession of the system at the same time. If, perchance, something of the kind took place, the symptoms would not follow the regular evolution. So, when a patient had constitutional syphilis, if a new chancre appeared to be hardened they would not find the glands swell, or the early manifestation of skin disease appear; and so of other symptoms. Superficial ulceration might take place, just as a spurious form of vaccination might arise on one who was still under the vaccine influence; but it was not a true case, it was not attended with the sequelæ. But if the constitutional disease were cured, if the syphilitic disposition were completely eradicated, then the patient would be able to contract a fresh indurated chancre, with all the subsequent symptoms. If this were the case—and he had observed it with great care, his experience dating back forty years—it proved that syphilis could be cured; and if syphilis could be eradicated, to ascertain whether a patient was cured or not when all the symptoms had disappeared there would be nothing else to do (though he knew that could not be done) but to try inoculation from an indurated chancre. If vaccination did not take, they were sure the vaccine disposition continued; if it did not continue, vaccination could take effect. In regard to syphilis, the proof had not been carried to this extent; but he had been able to observe that as long as the syphilitic influence continued, a patient could not contract an indurated chancre anew, and that, consequently, if cured, a new infection might take place. This was a great point gained in science, and it proved what he had said, that syphilis could be radically cured. Now, as to the treatment of the disease. As he had told them, Mr. Acton's ideas were completely his ideas, explaining his manner of treatment and his practice. He would first speak of the treatment of the first stage—that was to say, the primary sore. As soon as he had ascertained that there was a hardened chancre, with a swelling of the glands—not inflammatory, because the glands in this case never suppurated—he immediately instituted the mercurial treatment. There was one point on which there was some difference of opinion: many believed that it was impossible to prevent the accession of the secondary symptoms, the first manifestation of constitutional disease; many thought that no matter what treatment was employed the sequelæ would appear. Well, he had ascertained that if the treatment were soon begun and well carried through, the bursting out of the first secondary symptoms, the roseola, the swelling of the glands of the neck, etc., might be prevented. If this were not frequently the case it was because the treatment was resorted to too late, when the disease had had time to take root, and secondary symptoms were about to show themselves. In such cases it was not astonishing that secondary symptoms should appear, and the treatment ought not to be blamed; if the treatment were steadily continued they soon disappeared. But if the treatment were begun early, the observation of forty years gave him the assurance that secondary symptoms would not appear. When secondary symptoms had appeared, the best treatment was, as Mr. Acton had said, mercury. If they wished for a perfect cure, this treatment must be continued. In general it was not persisted in long enough; it was dropped as soon as the symptoms disappeared, or a short time after, and then it was not astonishing to see them reappear. But if the treatment were continued five or six months, having regard at the same time to sustaining the constitution in general, relapses would be found to be infrequent. He observed very few cases of relapse, and there would not be many when the treatment was well kept up—when the patient had patience enough, and the physician sufficient courage. After six months of that treatment and no symptoms reappearing, then the treatment with iodine must be begun, and continued for five or six months more. When a patient went to him, he said, "You will have a year's treatment—do you consent to that?" "Yes." "Very well; we will go on. If not, good bye." There were cases in which syphilis occurred in a healthy person—the only disease was syphilis. Then treatment was very easy—the case was a simple one; they had but one enemy to fight—all went on regularly. But, unhappily, in many instances syphilis was not alone; there was something else —scrofula, skin disease, scurvy, low constitution, poorness of the blood. They must understand that such complications as these altered the case; the treatment did not act so powerfully as it would do in the first case, as many of these complications were aggravated by the treatment. For instance, syphilis and scurvy might coexist —and the characteristic of the latter was poorness of the blood, while that of the former was a plastic condition of the blood. Here, therefore, was a counteracting influence to the treatment for syphilis. Now one thing must be known. Perhaps he was speaking too long? (No; go on.) Well, in many instances syphilis became the secondary consideration, and they must begin with the constitution of the patient, as debility was the disease that required first treatment. They must attack the strongest enemy first. Syphilis was sometimes quiet, and stopped and waited till they came to it. So, when they had improved the constitution, they might commence with the treatment, and they must begin by treating the constitutional complication. The best treatment was the proto-ioduret of mercury. The stomach bore this well in general. Sometimes it gave rise to a little diarrhœa, which was an easy thing to moderate; but when the stomach was not tolerant of the remedy, one capital treatment was that which Mr. Acton had told them he had confidence in—namely, rubbing-in. If this were not an unpleasant and disagreeable operation, certainly it would be in general about the best; he himself should prefer it. In rubbing-in, the action of the remedy was powerful and quick, and the stomach was not at all troubled with it. If it were not so disagreeable, and were a thing that could be done without being noticed, he should give it the preference. However, there were cases in which the skin was otherwise affected, in which there was a skin disease, and then friction could not be used. In a case of complication of syphilis and herpes rubbing-in could not be resorted to. In general, patients bore the iodide of potassium well, and in large doses. For his own part he frequently employed forty, sixty, eighty, even a hundred grains a day, and more. They must bear in mind that if they gave too small doses to some patients they would have no result; it was a remedy that passed through the body with great rapidity. He had had great experience of it, and he had found that in half an hour it had passed away in the urine. Iodide of potassium was a sort of broom of the blood. So they saw that the methodical treatment was this: mercury, iodide of potassium. But only one for the first stage, and only the other for the later stage of syphilis. No, the rule was absolute that as long as there were secondary symptoms well marked, mercury must be given; when there was a mixture of secondary and tertiary symptoms, mercury and iodide; for tertiary symptoms, iodide. To treat some patients with iodide would not advance them in any way. Why? Because there was frequently in the constitution, in the blood, something of the second stage, something that required the mercurial treatment. This might not show itself, but when iodide of potassium ceased to do good, the disease remaining stationary, let them go back to mercury again, and they would have a splendid result where they had thought there was no further possibility of curing the patient. This was what Mr. Acton had said, and he was completely and absolutely of Mr. Acton's opinion. But there was another thing. When syphilis had lasted for a long time, and had had a great effect on the constitution, it in some way disappeared, and left the patient with a complication existing that was not existing before. Sometimes a long course of treatment brought on a new disease—wasting of the constitution, poorness of blood. They must then stop all the specific treatment, and, applying themselves to the principal symptom, restore the constitution by preparations of iron, bark, tonics, and proper food, so bringing the patient back to the possibility of undergoing anew a regular methodical treatment, either by mercury or iodide, or a combination of these two remedies. In former times, when a person was thought to be syphilitic, physicians seemed unable to entertain any other idea than that of syphilis, and acted exclusively against a specific disease, neglected everything else, and in that way they experienced all the bad effects and accidental symptoms which a bad administration of the symptoms would produce. Mr. Acton had spoken of the use of bromide of potassium. His views were exactly the same as Mr. Acton's with respect to the use of the remedies at different stages, the necessity of having regard to the complications that might exist, and of dropping the treatment for awhile till the constitution was restored. This was regular and methodical, and his own manner of practice. But now, was bromide of potassium an anti-syphilitic remedy? He did not believe that it was. He might be mistaken; but he had experimented with it in syphilitic symptoms, and without any apparent result. But it was a splendid remedy in complications of syphilis. In some cases of symptoms referable to the nervous centres, bromide of potassium was an adjunct, and came to the help of mercury or the treatment by iodine. In some cases of brain disease with syphilis, and of disease of the spine or epilepsy, bromide of potassium did wonders. So that they would see it was a remedy to be applied in nervous complications that might occur, but they must not depend on it as an anti-syphilitic remedy. Now, there were symptoms following syphilis which were not syphilitic, and these must not be treated with mercury or iodide of potassium. For instance, there might be necrosis. Well, they could not bring a dead bone back to life, no matter what quantity of mercury or iodide of potassium they might give. A physician must know these things, and he (M. Ricord) ought almost to apologize for bringing them forward. It should be observed that specific remedies did not always act specifically. Certainly, there was no specific effect without a specific cause, but specific causes did not always act specifically. So there were some effects of syphilis, such as disease of the bones, that would afterwards act as a common irritant. In syphilis there might be an ulcerated bone in the nose or mouth, bringing on suppuration; mercury or potassium would not remove that, but let the diseased bone be removed, and the patient was frequently cured. They must take note of all these conditions—the nature of syphilis, the manner in which it conducted itself, its action on the constitution. Let them particularly take note that the general law of syphilis was the same as the general law of small-pox, vaccine, and measles. If they were sure of this from what he had said and from their own experience, then they might be sure that syphilis could be perfectly, radically cured. They could tell their patients that, and give them courage and hope. If the patient had courage to go through with the treatment, and the physician had courage enough to stick to it, the patient might be radically cured. He thanked them for the reception they had given him; it reminded him a little of his hospital in Paris.

A question was asked whether Dr. Ricord was a believer in salivation.

Dr. Ricord replied—No, surely not. Salivation was an accident following the treatment, and it must be avoided as much as possible. There was but one case in which he approved of salivation, and that was in disease of the eye—iritis. When this occurred, and salivation was brought on, the inflammation of the iris subsided.

Dr. Gross asked whether the soft chancre was capable of contaminating the constitution.

Dr. Ricord said his opinion was that a soft chancre, when accurately diagnosed, never gave rise to constitutional disease. This was a law as absolute as possible. But they must be careful, or errors of diagnosis might be made. It was not always easy to establish the difference between soft and hard chancre, but when the diagnosis was certain, they might be sure they would not have any constitutional disease after the soft chancre. On the contrary, even as long as six months after hard chancre secondary symptoms would appear. This was one of the most clearly established facts in practice. But the hardness of the chancre was not always well marked (*bien formulee*); it might be very superficial in those varieties that were attended with excoriation. When there was a something like parchment at the base, a chancre was very easily taken to be soft, but was not so; and he had had cases sent to him as instances of soft chancre which had been followed by secondary symptoms, but which were well characterized by the parchment-like base. However, there was a symptom of more value than the parchment base, a symptom that was one of the most important witnesses to constitutional affection, and that was the non-inflammation of the glands—they were cold and dull. In general several of them became enlarged; it was very seldom that only one was found to swell after hardened chancre; and not only were the glands swollen but the enlargement frequently occurred on both sides, in both groins. The enlargement of the glands was of much value as a characteristic of hardened chancre. The enlarged glands appeared very early, even during the first fortnight of the existence of the sore. With the soft chancre the glands did not always swell; in a great many cases there was no swelling. They would never find a real hard chancre without swelling of the glands; and they would also find many cases of soft chancre with swelling, these cases depending upon surgeons confounding the hard chancre with thickening dependent upon inflammatory infiltration of the tissue immediately around the sore. But if the glands should swell after soft chancre, it was probable that suppuration would come on. With hard chancre there was no inflammation and no suppuration. The older writers directed their efforts to cause an indurated sore to suppurate, in the belief arising from the practical observation that when a bubo suppurated there was no constitutional disease, and therefore they were under the belief that the poison was thrown out of the body. In their quaint way of putting the fact, "they did not like to shut up the wolf within the fold." But they could not bring on specific suppuration in the case of indurated glands; it was impossible. He had tried all means of doing it, and could not succeed in the cases of specific suppuration. In the instance of soft chancre what had they to do—await the occurrence of suppuration, which might either be attended by simply inflammatory or specific bubo? With the soft chancre the inflammatory bubo appeared sometimes two, three, or four weeks after the occurrence of the chancre, and it had the characteristic pus of the soft chancre. There was such a difference between hard and soft chancre that it was difficult to make a mistake. When a patient consulted him (M. Ricord) suffering from soft chancre, he said to him, " Be quiet; you may have a bubo; that will suppurate, but your constitution will be unaffected; you will not be liable to secondary symptoms." With a hard chancre he could predict indurated glands, attended by constitutional symptoms, within six months, provided proper treatment were not followed. He would add, that when it was decided that the case was one of hard chancre or soft chancre, the treatment was very simple. When there was a doubt as to the nature of the chancre, he waited till some characteristic symptom arose. But there were cases in which the existence of a soft chancre did not prevent a patient from contracting a hard chancre. The patient might have the two species at the same time, contracted from different sources. The two species, hard and soft chancres, do not depend upon the difference in the ground, but on a difference in the seed (*contagium*). So that the new comer who had relations with a woman suffering from the two species could take his choice. If the patient had a true indurated chancre and well diagnosed secondary symptoms, he might catch the soft chancre as often as he pleased, and it would be unattended with specific constitutional disturbance.*

Mr. Lord (London) asked Dr. Ricord what was his experience of municipal interference in respect to contagious diseases in Paris, and what was his opinion as to the effect of such interference in promoting immorality and degrading the character.

Dr. Ricord said it was surely a good thing to have the women examined. It made the disease less frequent—no doubt of it. From what had already been done in France he saw that the same practice would be beneficial here. It was already a great thing that English sailors no longer brought the disease into France; the French would take care it did not return back into England, and that was a free exchange.

The Chairman announced that the Council had resolved to make Dr. Ricord, Dr. Demarquay, and Professor Gross honorary members of the Association.—*London Lancet.*

* Dr. Ricord has established a law on which he sets great value, and for the verification of which he thinks the present and future generations will owe him a debt of gratitude. It is that of having discovered and described the UNICITY of the syphilitic diathesis—in fact, subjecting syphilis to the LAW which is common to small-pox, cow-pox, measles, etc.—ED. L.

